# Effect of menopausal hormone therapy on proteins associated with senescence and inflammation

**DOI:** 10.14814/phy2.14535

**Published:** 2020-08-28

**Authors:** Laura Faubion, Thomas A. White, Brett J. Peterson, Jennifer R. Geske, Nathan K. LeBrasseur, Marissa J. Schafer, Michelle M. Mielke, Virginia M. Miller

**Affiliations:** ^1^ Department of Surgery Mayo Clinic Rochester MN USA; ^2^ Robert and Arlene Kogod Center on Aging Mayo Clinic Rochester MN USA; ^3^ Department of Health Sciences Research Mayo Clinic Rochester MN USA; ^4^ Department of Physiology and Biomedical Engineering Mayo Clinic Rochester MN USA; ^5^ Specialized Center of Research Excellence on Sex Differences Mayo Clinic Rochester MN USA; ^6^ Department of Neurology Mayo Clinic Rochester MN USA

**Keywords:** estrogen, menopause, SASP, senescence‐associated secretory phenotype

## Abstract

**Background:**

Estrogen may inhibit cell senescence that contributes to age‐related disorders.

This study determined the effects of menopausal hormone treatments on circulating levels of markers of cell senescence.

**Methods:**

Growth differentiation factor 15 (GDF15), tumor necrosis factor receptor 1 (TNFR1), FAS, and macrophage inflammatory protein 1α (MIP1α) were measured in serum using multiplexed bead‐based assays and compared among menopausal women participating in the Kronos Early Estrogen Prevention Study randomized to either placebo (*n* = 38), oral conjugated equine estrogen (oCEE, *n* = 37), or transdermal 17β‐estradiol (tE2, *n* = 34). Serum levels of the senescent markers for each treatment were compared to placebo 36 months after randomization using the Wilcoxon rank sum test.

**Results:**

Serum levels of GDF15, TNFR1, and FAS, but not MIP1α, were lower in both the oCEE and tE2 groups compared to placebo. The difference in levels between treatment and placebo for GDF15, TNFR1, and FAS were greater for oCEE [−108 pg/mL (*p* = .008), −234 pg/mL (*p* = .0006), and −1374 pg/mL (*p *< .0001), respectively] than for tE2 [−76 pg/mL (*p* = .072), −105 pg/mL (*p* = .076), and −695 pg/mL (*p* = .036), respectively]. Additionally, TNFR1 showed a positive association with time past menopause (correlation = 0.255, *p *= .019).

**Conclusions:**

Circulating levels of some markers of cell senescence were lower in menopausal women treated with oCEE and tE2 compared to placebo. Differences in the magnitude of effect of the two active treatments may reflect the differences in circulating levels of estrogen metabolites due to formulation and mode of delivery. These data generate new hypotheses with regard to the effects of menopause on the biology of aging.

## Graphical abstract

Hormonal treatments reduced the levels of selected markers of aging in the blood of recently menopausal women.

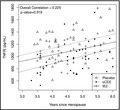

## 1 INTRODUCTION

Cell senescence, a state of cell cycle arrest due to the finite capacity of cells to proliferate, also occurs as a result of the accumulation of molecular and cellular damage (Hayflick & Moorhead, 1961). Senescent cells secrete an array of cytokines, chemokines, growth factors, and proteases collectively referred to as the senescence‐associated secretory phenotype (SASP) (Baker et al., 2011; Hoenicke & Zender, 2012). The array of SASP proteins includes many proteins that have been shown to be regulated by estrogen and to be secreted by platelets, leukocytes, and vascular endothelium including the metalloproteins (MMPs), tumor necrosis factor‐α (TNF‐α), ecosinoids, and serotonin (Miller et al., 2015; Raz et al., 2016; Raz, Hunter, Jayachandran, Heit, & Miller, 2014). However, other proteins considered to be part of the SASP array (e.g., GDF15, Fas, MIP1α, and TNFR1) may be more specific indicators of the systemic senescent cell burden (Schafer et al., 2020).

In response to DNA damage, senescence serves as an anticancer mechanism and may also have beneficial functions in embryogenesis, parturition, and tissue repair (Behnia et al., 2015; Demaria et al., 2014; Munoz‐Espin et al., 2013). However, senescent cells that are not cleared efficiently by the immune system disrupt tissue function, which increases the vulnerability to the onset and progression of a host of age‐related diseases, including pulmonary dysfunction, cardiovascular disorders, osteoporosis, neurodegeneration, and diabetes (Baker et al., 2011; Farr et al., 2019; Musi et al., 2018; Palmer et al., 2019; Schafer et al., 2017). In part, the deleterious effects of senescent cells are mediated by the SASP. Strategies to remove senescent cells and suppress the SASP are now being pursued as a means to counter age‐related diseases and geriatric syndromes (Baker et al., 2011; Kirkland & Tchkonia, 2017; Schafer et al., 2020).

Estrogen is a steroid hormone implicated in modulating cell senescence. For example, estrogen decreases cell senescence in endothelial progenitor cells, and activates estrogen receptor alpha (ERα) to inhibit cell senescence‐like phenotypes in human epithelial cells (Liu et al., 2016). Estrogen also slows deficits associated with aging and cell senescence in bone, such as declining bone density (Farr et al., 2019). However, little is known regarding how cell senescence might be modified by natural changes in hormone concentrations, such as those that occur during menopause, and how this might be modulated by hormone therapies. Over 20% of the U.S. population will be aged 65 years or older by the year 2030 so it is important to better understand and address the health problems associated with aging (Vespa, Armstrong, & Medin, 2018). Therefore, this study examined whether menopausal hormone therapies, in the form of oral conjugated equine estrogens (oCEE) and transdermal 17β‐estradiol (tE2), altered the circulating levels of a specific set of SASP proteins in women who had undergone natural menopause. It was hypothesized that hormone therapies would decrease the circulating concentrations of specific SASP proteins.

## 2 METHODS

### 2.1 Study design

Serum samples collected from a subset (*n* = 109) of women who participated in the Kronos Early Estrogen Prevention Study (KEEPS) were used to evaluate the effect of menopausal hormone treatments (HT) on markers of cellular senescence. KEEPS was a placebo‐controlled, double‐blind randomized trial to assess the impact of HT on progression of atherosclerosis and carotid intima‐medial thickening. KEEPS participants were between the ages of 42 and 58, within 5–36 months of their last menstrual period. Full inclusion and exclusion criteria are listed in the clinical trial description (KEEPS; NCT00154180) (Harman et al., 2005). Women were randomized to placebo patches and pills (*n* = 38), 0.45 mg/day oCEE (*n* = 37), or 50 µg/day tE2 in a weekly patch (*n* = 34). Women assigned to active treatments were also given micronized progesterone (200 mg/day) for the first 12 days of each month. Treatment continued for 48 months with a fasting venous blood sample collected each year. Blood was collected according to a protocol that minimized the activation of platelets (Jayachandran, Miller, Heit, and Owen (2012). Serum prepared from the 36‐month blood sample from a subset of the KEEPS participants was used in this study as this sample was collected during the progesterone treatment phase of the month. Serum was frozen at −70°C, and analyzed after one thaw. Perliminary experiments utilized a broad plateform of proteins considered in the SASPs including sclerostin protein (SOST), disintegrein and metalloproteinase with thrombospondin type 1 motifs, member 13 (ADAMTS13), and chemokine (C‐C motif) ligand 17 (CCL17 orTARC). However, many measurements were below the detection limits of the assay, thus limiting statistical power upon which to draw meaningful conclusions. Therefore, the selected set of SASP proteins represents those whose measurements were robust and proteins that had not been previously shown to be regulated by estrogen in plasma or platelet lysate in recently menopausal women of KEEPS (Miller et al., (2015).

### 2.2 Assays

Multiplexed bead‐based immunoassays (Magnetic Luminex Assay, Human Premixed Multi‐Analyte Kit, R&D Systems) were used to measure the presence of growth differentiation factor 15 (GDF15), tumor necrosis factor receptor 1 (TNFR1), FAS, and macrophage inflammatory protein 1α (MIP1α, also referred to as CCL3). All assays were performed according to the manufacturer's protocols (Schafer et al., 2020). Sensitivities and intra‐ and inter‐assay confidence values of each protein in the assays are listed in Table 1. The assays were executed according to the protocols provided by the manufacturer.

**Table 1:** Sensitivity of Assays for SASP proteins in pg/mL, confidence values for assays in percent

Protein	Sensitivity (pg/mL)	InterAssay Confidence Value	IntraAssay Confidence Value
			
MIP‐1 α	16.2	6.8%	12.9%
GDF15	1.2	10.7%	14.7%
TNFR1	41.0	6.2%	9.1%
Fas	3.2	5.5%	8.1%

The samples were deidentified during data collection and analysis. Samples were placed such that all treatment groups were represented on each plate in order to mitigate possible variation due to difference among plates.

### 2.3 Statistical methods

Descriptive statistics are provided as mean (standard deviation) and median (quartile 1, quartile 3). Kruskal–Wallis tests were used to test overall differences in demographics and protein levels across the three treatment groups. Pairwise comparisons of tE2 versus placebo, and oCEE versus placebo were conducted using Wilcoxon rank sum tests with a Bonferroni adjustment for multiple comparisons. Hodges–Lehmann estimates of protein level location shifts are given for significant differences. Associations of potential confounders [age, body mass index (BMI), 36 month visit age, and time since menopause] with protein levels were tested using Spearman correlations.

## 3 RESULTS

**Figure 1:**
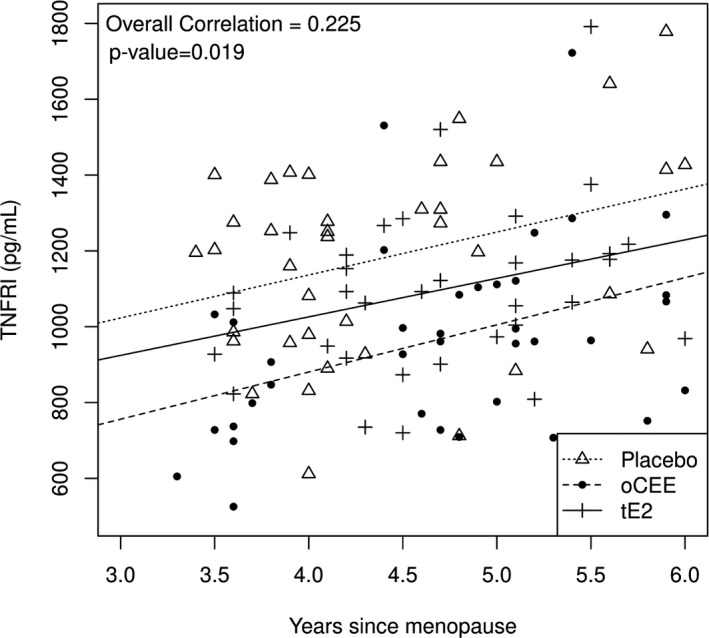
Correlation between TNFR1 levels in serum (pg/mL) and years past menopause. Each point represents an individual from the randomized groups: oCEE (*n* = 37), tE2 (*n* = 34), and placebo (*n* = 38) treatments

The average age of the 109 women at enrollment was 53.1 years, and 56.1 years at the 36‐month visit. Average time since menopause at the 36‐month visit was 4.6 years with a range of 4.0 to 5.1 years; average BMI was 27.0 kg/m^2^ (range of 24.2 to 30.8 kg/m^2^). There were no differences in demographics (age, years since menopause, BMI) across the three treatment groups at 36 months (Table 2). Age at baseline and at the 36‐month visit, BMI, and time since menopause were not significantly correlated with protein levels, except that higher TNFRI levels were associated with longer time since menopause (Figure 1).

**Table 2:** Demographic characteristics by treatment group, presented as mean (Standard Deviation)

	Placebo	oCEE	tE2	*p*‐value
	*N* = 38	*N* = 37	*N* = 34	
Age (years) at 36‐month visit	55.8 (2.4)	56.2 (2.4)	56.2 (2.4)	.2825
Years since menopause	4.4 (0.8)	4.7 (0.8)	4.7 (0.7)	.1213
Body Mass Index (kg/m^2^)	27.1 (3.7)	27.7 (4.6)	26.3 (4.3)	.3794
Estradiol (E2)	20.7 (7.6)	22.9 (6.2)	23.4 (13.2)	.2071

Abbreviations: oCEE, conjugate equine estrogens; tE2, transdermal 17β‐estradiol.

**Figure 2:**
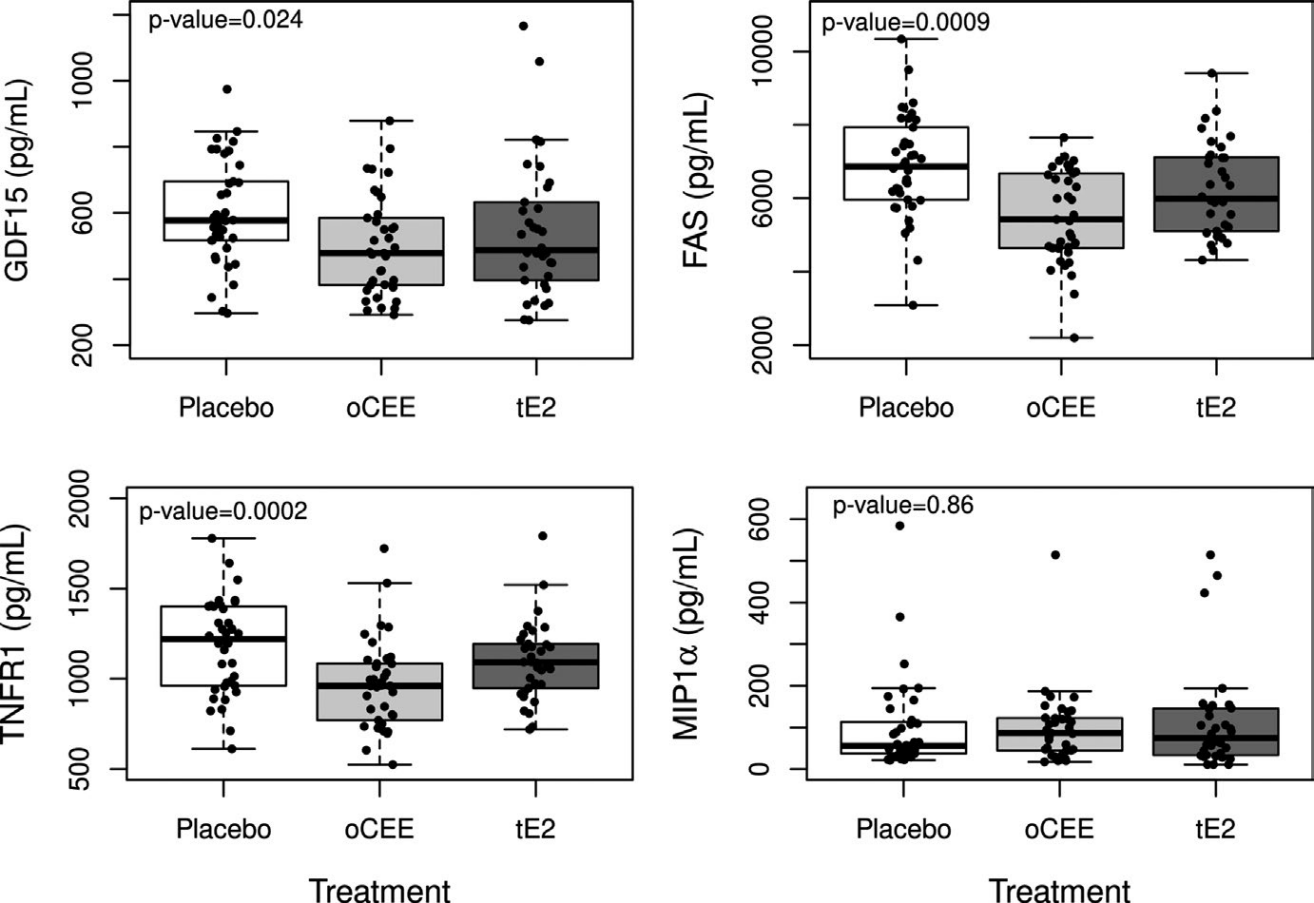
Protein levels in pg/mL for GDF15 (upper left), Fas (upper right), TNFR1 (lower left), and MIP‐1α (lower right) for oCEE (*n* = 37), tE2 (*n* = 34), and placebo (*n* = 38) treatments. Data are shown as medians, solid line; interquartile ranges, boxes; standard deviation, lines and bars

At 36 months, serum levels of GDF15, TNFR1, and Fas but not MIP1α varied by treatment group, with significantly lower levels in both the oCEE and tE2 group compared to placebo (Figure 2). GDF15, TNFRI, and FAS level difference estimates (95% CI) were −108 (−180, −31), −234 (−348, −120), and −1374 (−1940, −711) pg/ml respectively (all adjusted p‐values < 0.02) for the oCEE versus placebo groups (Table 3). Levels of the measured SASP proteins did not correlate with circulating levels of either estrone or 17β‐estradiol (data not shown).

**Table 3:** Protein levels by treatment group at 36 months, adjusted for years since menopause

	Treatment	*N*	Mean (*SD*)	Median (Q1, Q3)	3‐group *p*‐value	*p*‐value versus placebo^a^
GDF15	Placebo	37	598.4 (158.1)	577 (517, 695)	.0141	—
	oCEE	38	498.8 (152.8)	479 (383, 585)	—	.0022
	tE2	34	543.6 (207.3)	487 (396, 633)	—	.2104
TNFRI	Placebo	37	1,181.7 (261.3)	1,220 (962, 1,401)	<.0001	—
	oCEE	38	967.3 (248.3)	961 (771, 1,084)	—	<.0001
	tE2	34	1,096.5 (217.9)	1,091 (949, 1,193)	—	.0680
Fas	Placebo	37	6,871.3 (1,411.1)	6,864 (5,962, 7,931)	<.0001	—
	oCEE	38	5,505.1 (1,266.6)	5,424 (4,649, 6,675)	—	<.0001
	tE2	34	6,256.9 (1,255.9)	5,989 (5,105, 7,119)	—	.0552
M1P1 α	Placebo	36	100.3 (111.6)	56 (38, 114)	.8411	—
	oCEE	35	98.6 (87.9)	87 (44, 123)	—	1.0000
	tE2	33	113.0 (124.5)	75 (34, 145)	—	1.0000

Abbreviations: oCEE, conjugate equine estrogens; tE2, transdermal 17β‐estradiol.

^a^ Bonferroni‐corrected for multiple comparisons.

## 4 DISCUSSION

Serum levels of selective senescence‐associated proteins (GDF15, TNFR1, and Fas) were lower in healthy, recently menopausal women using HT for 36 months compared to placebo. Furthermore, oCEE had a greater impact on reducing circulating levels of the SASP proteins than did tE2. Although GDF15, TNFR1, Fas, and MIP1α have demonstrated sensitivity to age, they were not correlated with age at baseline and 36 months in this study, likely due to the small age range of participants (Schafer et al., 2020). GDF15, TNFR1, Fas, and MIP1α were selected from a large set of analytes because they have been shown, along with the SASP proteins osteopontin, Activin A, and interleukin 15, to be predictive of adverse health events and had not previously been evaluated as constituents of the cytokine mileu affected by menopausal HT (Miller et al., 2015; Raz et al., 2014, 2016; Schafer et al., 2020).

The SASP includes proteins with diverse biological functions and their production may vary by the senescent cell of origin. GDF15, a cytokine belonging to the transforming growth factor (TGF)‐ β superfamily, is produced by active macrophages and is secreted by human senescent endothelial cells (Bootcov et al., 1997; Schafer et al., 2020). In humans, GDF15 is expressed in response to cellular damage and serum levels are positively correlated with age (Schafer et al., 2020). GDF15 also associated with senescence to promote colon cancer formation, and human airway epithelial senescence (Guo et al., 2019; Wu et al., 2016). However, secretion of this protein by senescent vascular endothelial cells seemed to be beneficial by promoting function of vascular progenitor cells (Ha et al., 2019). In men with coronary artery disease, GDF15 levels were inversely correlated to serum testosterone and testosterone/estradiol ratio, but not to estradiol. However, in the same study, estradiol significantly decreased GDF15 expression in vitro (Liu, Dai, Cui, Lyu, & Li, 2019). This study supports the effect of estradiol on GDF15 expression in women who were at low risk for cardiovascular disease. Estrogen could function to inhibit the increase in GDF15 that occurs with age.

TNFR1, a receptor that modulates the actions of tumor necrosis factor alpha, increases cytotoxicity of a cell and causes the production of other inflammatory proteins (Tartaglia & Goeddel, 1992; Vandenabeele, Declercq, Vanhaesebroeck, Grooten, & Fiers, 1995). TNFR1 is modulated by estrogen (Deb et al., 2004). The modulatory effect of estrogen on expression of TNFR1 may be dose‐dependent as high concentrations of estrogen upregulated the expression in cancers, whereas in this study TNFR1 levels decreased with menopausal HT. TNFR1 is also associated positively with frailty and with rehospitalization after surgery (Schafer et al., 2020). The correlation between TNFR1 levels and age past menopause suggests the involvement of yet other unidentified factors in regulation of this receptor. The ligand for this receptor, TNF‐α, decreased in platelet lysate with HT over the 48th month of treatment. However, there were no differences in the plasma levels of this ligand among groups (Miller et al., 2015). The distinctions in platelet content and plasma levels of the ligand suggest that other cells may contribute to the circulating pool of this cytokine, some of which may be at various stages of activation of senescence. The relationship betwen ligand–receptor regulation by estrogen remains to be explored.

The Fas protein is a key regulator of the apoptotic cell pathways. Apoptosis is an essential cell mechanism in the maintenance of tissues. Estrogen inhibits the Fas/FasL system through ERα (Mor, Straszewski, & Kamsteeg, 2002). Regulation of apoptosis is important in the treatment of age‐related diseases (Muradian & Schachtschabel, 2001), and hormone treatments could potentially facilitate these processes in menopausal women. This finding further supports the hypothesis that Fas is involved in aging processes and is impacted by estrogen.

Other cytokines and chemokines implicated in the SASP, including MIP1α, may be inhibited by estrogen treatment (Matejuk et al., 2001). The lack of an effect on MIP1α levels in this study may reflect the small number of participants in each group, the low levels of E2 reached with the treatment, or that the presence of progesterone may have antagonized the effects of the estrogen. The lack of correlation of serum levels of hormones with those of the SASPs proteins may reflect the narrow range of serum hormone values or that sensitivity of the regulatory mechanisms for the SASPs at the cellular level may differ by tissue as the tissue or cellular origin of the SASPs cannot be determined by the study approach. Additional research is needed to clarify these relationships.

Functionally, SASPs correlate positively with frailty and adverse cardiovascular surgical and cancer outcomes (Schafer et al., 2017). The results of this study suggest that HT in menopausal women may reduce frailty. Although specific measures of frailty were not measured in KEEPS and the women were relatively young (mean age 55 years), some chronic conditions of aging, [decreases in bone mineral density, sleep disturbances, depression, vaginal atrophy, and deposition of β amyloid in the brain], were alleviated by the HT treatments (Cintron et al., 2017; Farr, Khosla, Miyabara, Miller, & Kearns, 2013; Gleason et al., 2015; Kantarci et al., 2016; Taylor et al., 2017). In older women of Estrogen Early versus Late Intervention with Estadiol (ELITE), estrogen treatments reduced the rate of increase of carotid intima‐medial thickness (Hodis et al., 2016), a result consistent with the finding of this study that estrogen lowered the endothelial‐prominent GDF15 SASP. On the contrary, abrupt loss of ovarian hormones by bilateral oophorectomy prior to the age of 45 years increased the risk for age‐related morbidities in women (Rocca et al., 2016, 2017, 2018; Zeydan et al., 2019). Indeed, many of the SASPs were present in older cohorts and women who had undergone oophorectomy for ovarian cancer (Schafer et al., 2020). Taken together, these studies support the hypothesis that ovarian hormones and HT at menopause modulate the aging process. Indeed, the results are consistent with previous literature demonstrating an inhibitory effect of estrogen on cellular processes associated with cellular inflammation, apoptosis, and senescence (Evans, MacLaughlin, Marvin, & Abdou, 1997; Fliegner et al., 2010; Imanishi, Hano, & Nishio, 2005a; Imanishi, Kobayashi, Hano, & Nishio, 2005b; Le May et al., 2006; Liu, Guo, & Guo, 2002; Miller, Jayachandran, Hashimoto, Heit, & Owen, 2008; Spyridopoulos, Sullivan, Kearney, Isner, & Losordo, 1997; Turner & Kerber, 2017). Direct evidence for regulation of organ/cells specific SASPs by estrogenic hormones requires further study with particular attention to the chronological age, menopausal age, use of menopausal HT, source of the sample (plasma or serum, as the relative concentration of some proteins may be influenced by platelet activation of bound to factors activated by the coagulation cascade.

Differentiation of various formulations and metabolites of estrogen on tissue‐specific cellular mechanisms of aging warrants further investigation, especially if some of these factors are to be considered as potential senolytics (Stout et al., 2017; Yanai & Fraifeld, 2018). It is not possible from this study to identify which steroid metabolite was most effective on specific target cells. CEE consists of a mix of estrone, estrone sulfate as well as 17β‐estradiol, which have antioxidant properties and differentially bind to estrogen receptors (Bhavnani & Stanczyk, 2014). The first past metabolism of some of the components of CEE to 17β‐estradiol varies among women due, in part, to genetic variants in enzymes associated with steroid metabolism, including sulfonation and cellular uptake (Moyer et al., 2018; Moyer, de Andrade, Weinshilboum, & Miller, 2016). Therefore, the greater effectiveness for oCEE compared to tE2 could reflect direct effects in the liver. It remains to be tested if oral formulaltions of 17β‐estradiol would have similar effects on these markers of senescence as oCEE. Taken together, these results could indicate a new approach by which to study and determine the effects of HT on age‐related diseases.

## CONFLICTS OF INTEREST

The authors do not have any financial conflicts of interest to declare.

## AUTHOR CONTRIBUTIONS

Faubion conducted the assays, analyzed the data, and drafted the paper; White supervised the assays and assisted in interpretation of the data; Peterson and Geske were involved in the statistical analyses of the data; LeBrasseur, Schafer, Mielke and Miller conceptualized study, assisted in interpretation of the data, edited the manuscript, and provided funding for the project.
